# The Effect of Parenting Quality on Child Development at 36–48 Months in China’s Urban Area: Evidence from a Birth Cohort Study

**DOI:** 10.3390/ijerph17238962

**Published:** 2020-12-02

**Authors:** Xihong Wu, Gang Cheng, Cai Tang, Qunhui Xie, Simin He, Ruotong Li, Yan Yan

**Affiliations:** Department of Epidemiology and Health Statistics, Xiangya School of Public Health, Central South University, Xiangya Road 110, Changsha 410078, China; wuxihong@csu.edu.cn (X.W.); gangcheng@csu.edu.cn (G.C.); tang_caill@163.com (C.T.); xiequnan@163.com (Q.X.); hesimin1969@csu.edu.cn (S.H.); liruotong@csu.edu.cn (R.L.)

**Keywords:** parenting quality, child development, cognitive development, suspected development delay

## Abstract

Environmental exposures, especially parenting quality, are critical for later child development. This study aimed to determine the status of parenting quality and suspected development delay of preschool children in China’s urban area and explore the associations between these two factors. The research was based on a birth cohort study conducted in Changsha, Hunan province, China. We used the Parenting Assessment Tool and Ages and Stages Questionnaires, Third Edition (ASQ-3), to measure parenting quality and child development status, respectively. Other data were collected from maternal health manuals and self-administered questionnaires during the follow-up period. The generalized estimating equation was used to examine whether parenting quality was significantly associated with child development outcomes. In the study, good parenting quality was 33.6% measured at 18 months, and suspected development delay was below 10% at 36–48 months among urban China; we observed negative associations between parenting quality scores and child development scores; poor parenting quality had a negative association with suspected development delay [OR and 95% CI: 2.74 (1.17, 6.40)], girls [OR and 95% CI: 0.33 (0.16, 0.69)] and maternal education years (>12 years) [OR and 95% CI: 0.27 (0.12, 0.64)] were protective factors for suspected development delay. Our findings highlighted the importance of good parenting quality among children in urban areas of China through a birth cohort study and may be used to reduce the children at high risk of developmental delay as a future intervention program.

## 1. Introduction

Early childhood, especially the first two years in early life, is a critical period for rapid growth and cognitive development [1]. Black et al. indicated that 250 million children (43%) younger than 5 years in low-income and middle-income countries are at risk of not reaching their developmental potential, and the number is as high as 17.43 million in China [2,3]. Cognitive development in early childhood is attributed to genes and the environment [4]. Genetic influences on cognitive development occur through a transactional process, in which genetic predispositions lead children to evoke cognitively stimulating experiences from their environments [5]. In low-income and middle-income countries, individual differences in environments have a greater influence on IQ and genetics less so [6]. Child cognitive development depends on multiple factors, which interact with each other and can be mutually reinforcing the process of development [2,7,8,9]. Disadvantaged exposures and experiences in early childhood increase the risk of low social, cognitive, and health outcomes, and these outcomes are increasingly challenging to reverse beyond early childhood [10]. 

According to the Integrated Maternal and Child Health Development (IMCHD) project in China [11], the prevalence of suspected developmental delay in children younger than three years in China poverty-stricken areas was 39.7%. It also emphasized that parenting played an essential role in the suspected development delay. According to a randomized comparison trial, the children who received encouraging parenting behaviors had higher development scores than the control group [12]. Disadvantaged experiences in early childhood have lasting effects on cognitive outcomes [13], but higher parenting quality predicted better performance after children suffered from brain injuries in early childhood [14]. Most of the current studies focusing on child development are cross-sectional studies and lack longitudinal study evidence [15,16] and could not infer causal and temporal associations. Due to the regional economic development disparities, most studies were conducted in poor rural areas in China and less in urban areas [11,17]. The effect of family rearing quality on cognitive development differs in diverse economic conditions [18]. A longitudinal birth cohort study showed a stronger association of child cognitive development and socioeconomic status compared to measures of other home environmental determinants (parenting) in low- and middle-income areas [19]. We need to conduct surveys in China’s urban areas to find an association between parenting quality and child development.

To address these research gaps, we conducted a cohort study in urban areas of Hunan province. This study aimed to determine the status of parenting quality and suspected development delay of the children at 36, 42, 48 months, explore the associations between these two factors and provide evidence for future intervention to reduce suspected development delay.

## 2. Materials and Methods

### 2.1. Study Design and Participants 

This study was based on an in-progress birth cohort project, which is being conducted in three communities in the Kaifu District of Changsha in Hunan province from January 2015 to December 2020. The inclusion criteria for the research included: (1) mothers and infants were permanent residents in the Kaifu District; (2) infants had healthcare records in the Community Health Management Information System (CHMIS); (3) mothers or infants’ caregivers agreed to participate and signed the written informed consents. The exclusion criteria included: (1) mothers had a history of mental illnesses or brain diseases; (2) the newborn suffered from a severe medical condition. 

**Figure 1:**
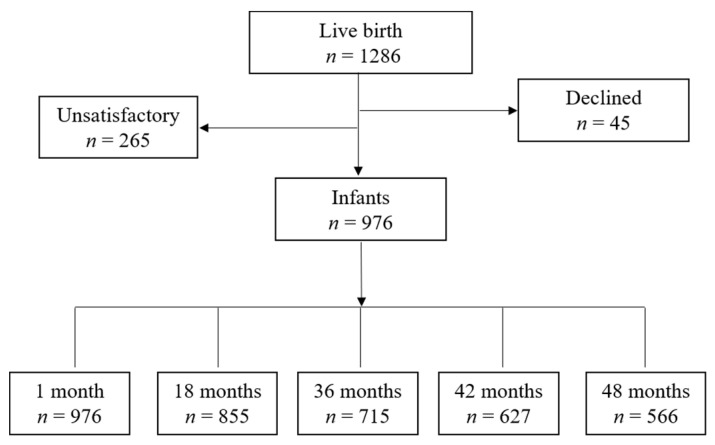
Participant study flow.

Every live birth at three communities was invited to participate in the study (*n* = 1286) from January to December 2015. According to the inclusion and exclusion criteria, the cohort recruited 976 mother–offspring pairs (Figure 1). Data were collected through the maternal health manual, CHMIS, and a self-administered questionnaire that was pretested in a pilot study. The follow-up visits were performed at the ages of 1, 3, 6, 8, 12, 18, 24, 36, 42, and 48 months. 

We collected the development data at 36, 42, and 48 months, parenting quality data at 18 months, and the baseline data at 1 month. Figure 1 describes the number of participants at each time point. The study was approved by the Independent Ethics Committee Institute of Clinical Pharmacology, Central South University, Changsha, China. (Project number: CTXY-130041-3-2).

### 2.2. Measures

#### 2.2.1. Children Development Outcomes 

ASQ-3 was used to measure preschool children’s development outcomes at 36, 42, 48 months. ASQ-3 is a parent-completed questionnaire used as a general developmental screening tool to identify children’s potential developmental problems from 1 to 59 months [20]. It has excellent psychometric properties, with high reliability and validity in China [21]. The ASQ-3 is a developmental screening instrument consisting of 21 intervals, different child’s age group has a corresponding one. Each questionnaire includes 30 items in five domains: communication (CM), gross motor (GM), fine motor (FM), problem-solving (CG), and personal social (PS). Each “yes” item is scored 10 points, “sometimes” is scored 5 points, and “not yet” is scored 0 points, and the sum score of each domain is 60. We compared the sum scores of every domain with the national normative cutoff point of China, then regarded children whose scores were lower than the cutoff point of the national norm in any field as suspected developmental delay.

#### 2.2.2. Parenting Quality 

We used the parenting assessment tool (PAT) to assess the parenting quality and skill at 18 months. PAT is a reliable and marginally valid measurement tool for determining Chinese urban parents [22]. Moreover, PAT referred to the content, structure, and scoring method of Home Observation for Measurement of the Environment (HOME). The questions were designed to be broad, to elicit the general parental attitudes about parenting. PAT includes 8 factors: parenting concepts, acceptance, parent–child relationship, learning material, language stimulation, outside activity, feed, and safety. Each element has 4–5 items; the answer of each item as “yes” is scored 1 point, “no” is scored 2 points, and “not clear” is scored 3 points. The higher the PAT score was, the fewer parents knew about parenting. When all items’ answers were “yes,” the parenting quality was considered good parenting quality and vice versa.

#### 2.2.3. Confounding Factors 

Based on previous research [8,23,24,25], we covered multiple potentially confounding factors in our analysis, including child gender, child order, family socioeconomic factor, delivery mode, and paternal smoking status during pregnancy. Child order was classified into 2 groups: less than 2 children, more than or equal to 2 children. Family socioeconomic factors contained family income and parental education. Family income was measured by 4 groups: less than 2000 Chinese Yuan (CNY), 2000–4999 CNY, 5000–9999 CNY, and more than 10,000 CNY. Parental education was divided into 2 categories: less than or equal to 12 years and greater than 12 years. Delivery modes included vaginal and cesarean delivery. The paternal smoking status was classified as smoking and not smoking during pregnancy.

### 2.3. Statistical Analysis

Cohort characteristics for participant demographics were presented as mean (SD) for numerical variables *n* (%) for categorical variables. A generalized estimating equation (GEE) model was applied to find the correlation between parenting quality and developmental outcomes at different follow-up times from the same child. We constructed two kinds of GEE models to describe the associations. In the first model, the scores in different domains of ASQ-3 and the scores in 8 factors of PAT as dependent and independent variables were quantitative variables. In the second model, dependent and independent variables were qualitative variables. Suspected development delay was the dependent variable, and independent variables were parenting quality and other factors. The potential factors in both analyses were child gender, family income, parental education, delivery mode, child order, and paternal smoking during pregnancy [23,26,27]. The above analyses were performed in SPSS version 22 (IBM, New York, NY, USA). 

The number of respondents varied across waves (Figure 1). Most of the missing data were due to wave no-response (move and exit follow-up). Of the 976 respondents, 246 did not respond to the variables of interest (ASQ-3) in this study, so we removed these respondents. Besides, 538 respondents provided data on all three waves, 102 on two waves, and 90 on one wave. Altogether, 730 respondents provided data on ASQ at least once. There was no statistical difference in the general characteristics of the included and excluded subjects. The analysis was based on these 730 respondents. We used the “MissMech” function to test missing data; the result indicated that missing data were non-systematic [*p* = 0.730], which supported the multiple imputations. We completed quantitative and qualitative variables by “mice” package; the method was a random forest model. Missing data analysis was calculated on the R 3.5.1 version.

## 3. Results

Table 1 summarizes participant demographics among the included 730 children. We got all the information of gender, and boys were 373 while girls were 357. Most families in the study were single-child families (71.9%). About a quarter of the families (27.8%) had 2 children or more. According to the delivery method, 59.1% were vaginal delivery, and 40.65% were cesarean delivery. The constituent ratios of family income categories were 3.4%, 51.5%, 37.8%, and 4.2%. A total of 617 (84.5%) mothers had more than 12 years of education; the number of fathers was 622 (85.2%). About 52.7% of fathers smoked during pregnancy. A third of families (224) provided good parenting quality, while 443 children had poor parenting quality.

**Figure 2:**
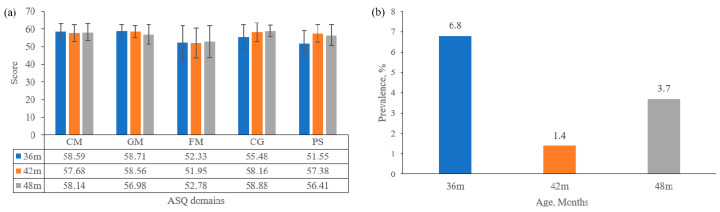
Children development outcome at 36 months, 42 months, 48 months. (**a**) ASQ-3 scores in different domains of children; (**b**) suspected developmental delay at 36 months, 42 months, 48 months.

**Figure 3:**
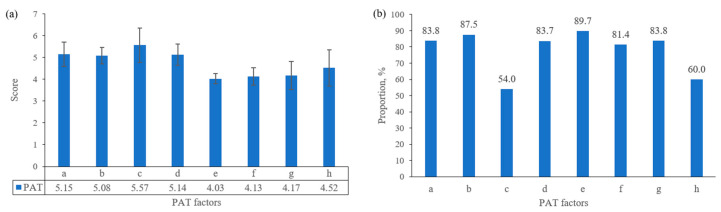
Parenting quality at 18 months of children. (**a**) PAT scores of different facts; (**b**) proportion of good parenting quality at 18 months. a. parenting concepts; b. acceptance; c. parent–child relationship; d. learning material; e. language stimulation; f. outside activity; g. feed; h. safety.

Figure 2 was a descriptive analysis of ASQ at 36 months, 42 months, 48 months. As shown in the figure, each domain’s mean scores were higher than 50, and the FM domain had the lowest scores in three check times. The prevalence of suspected development delay was 6.8% at 36 months, 1.4% at 42 months, and 3.7% at 48 months. Figure 3 describes parenting quality at 18 months of children, including PAT scores and the proportion of good parenting quality in each factor. The proportion of good parenting quality in each factor was from 54% to 89.7%; those in most factors were higher than 80%. The lowest proportion of good parenting was in the parent–child relationship (54%); the highest proportion was in language stimulation (89.7%). 

Table 2 presents the associations between parenting qualities and children’s cognitive development. The results showed us that if parents knew more about parenting, children’s ASQ scores would be higher, and different PAT factors affected the ASQ domains. As shown in Table 2, children’s CM scores would be higher if parents knew more about parenting concepts, parent–child relationships, language stimulation, and children’s safety. Poor parenting qualities in attention, communication, learning material, and language stimulation had adverse effects on children’s GM scores. FM scores were higher when scores of parenting concepts and learning material were closer to 5 points. Parental feeding concepts were negatively correlated with CG. We found a negative correlation between attention, parent–child relationship, the outside activity of PAT, and PS of ASQ.

Table 3 provides the results of factors influencing suspected development delay. The significant factors include parenting quality, child gender, and maternal education. Compared with children under good parenting quality, children under poor parenting quality had a higher prevalence of suspected development delay [OR and 95% CI: 2.74 (1.17, 6.40)]. The girls were less likely to suffer from suspected development delay than boys [OR and 95% CI: 0.33 (0.16, 0.69)]. Maternal education years more than 12 is a protective factor for suspected development delay among the observations [OR and 95% CI: 0.27 (0.12, 0.64)].

## 4. Discussion

In the study, we found that the proportion of good parenting quality in urban China was 33.6% measured at 18 months. The prevalence of suspected development delay was 6.8% at 36 months, 1.4% at 42 months, 3.7% at 48 months. We also observed the negative association between parenting quality and communication, gross motor, fine motor, problem-solving, and personal-social of children; poor parenting quality increased the risk of suspected development delay; girls and maternal education years (>12 years) were the protective factors for suspected development delay. 

Our study revealed that the prevalence of suspected development delay was below 10% at 36–48 months among urban China. According to a survey about suspected development delay, the prevalence of suspected developmental delay among children aged 6–35 months was 35.7% in poor rural areas of China [28], 17% in Senegal, and 24% in Brazil [29]. Other studies’ higher prevalence may reflect the environmental and nutritional factors, including socioeconomic status, contributing to disparities in child development [30]. The scores of FM were the lowest among five domains of ASQ, which suggested the need for further intervention of fine motor in an early lifetime. In our study, good parenting quality was 33.6%, and poor parenting quality was 66.4% at 18 months. According to the IMCHD project in rural China, children’s good care quality was about 25%, poor and medium parenting quality was about 75% at 12–23 months [31]. The different proportions were likely due to positive associations between socioeconomic classifications and the availability of children’s books and playthings.

We observed negative associations between parenting quality scores and child development scores, similar to the previous study. A longitudinal birth cohort in Canada has revealed the protective effects of parent–child interactions and language stimulation on child language development [32]. An adequate home environment, which represents good parenting quality, is associated with better child motor development in southern Brazil [33]. Some randomized controlled trials had highlighted that interventions on maternal play and parenting skills have also improved young children’s social, emotional, communication, language, and cognitive competence [25,34,35]. Our multivariable analysis confirms these findings, and improving the quality of different parenting behaviors is a feasible and effective way to enhance child development in primary community health services.

In public health, the development of effective intervention strategies requires an understanding of risk factors. We used the GEE model to testify parenting quality and confounders affecting suspected development delay. Poor parenting quality was a risk factor of suspected development delay, which was consistent with previous studies. A pregnancy cohort has highlighted that nondaily parent–child interaction increased the risk of delay [36]. Child gender, as an essential demographic characteristic, may play a role in child development [37]. For example, boys were statistically significant predictors of low cognitive development, according to an American birth cohort study [38]. In our study, we found that gender impacted child development; girls got better outcomes than boys. A meta-analysis of parent–child language interactions showed that mothers talk more to their daughters than their sons [39]. Thus, more maternal language interaction preference for girls than boys could explain this result. We found that maternal education affected suspected development delay, and family income had no effect on child development among socioeconomic variables. Mothers with higher education levels may have more knowledge of parenting, pay more attention to children’s cognitive development, and improve their cognitive development through scientific methods [40,41]. The result of family income may be that the role of socioeconomic status on children’s development gradually decreases with the development of social income [6,19]. We observed that paternal education was not statistically associated with suspected development delay. One possible explanation may be that limited father participation was insufficient to show positive child development outcomes. The result suggested that intervention strategies should target different child gender and focus on parenting quality to avoid adverse developmental outcomes.

To our knowledge, this is one of the first longitudinal studies investigating the impact of parenting quality on child development in urban China. The study population was representative of the urban population in Hunan. We randomly selected three communities of the Kaifu District as our study sites. However, the present study was subject to certain limitations. First, there exists evidence that parenting quality would change over time; one explanation may be the bidirectional nature of parenting behavior and child development [27,31]. Further evidence from cohort studies and interventional studies is needed to consider the change in parenting quality. The second limitation is that the self-administered questionnaire was not assessed for validity and might result in some inaccurate information. Another limitation was that ASQ is only a screen tool for suspected developmental delay. The potential bias caused by misclassification error should be considered when interpreting the findings.

## 5. Conclusions

The prevalence of child development in China’s urban areas is lower than in China’s poor rural areas, and parenting quality is higher than in rural areas. Diverse parenting factors influence different development outcomes, and the parenting quality is associated with suspected development delay. For a single development domain, we could have interventions individually to prevent children from suspected development delay. Strategies targeting parenting quality among children in China’s urban areas should be addressed for a future intervention program to reduce the children at high risk of developmental delay.

## Figures and Tables

**Table 1:** Participant demographics among the included 730 children.

Characteristics	*n* (%)
Child gender	
Boys	373 (51.1%)
Girls	357 (48.9%)
Child order	
<2	525 (71.9%)
≥2	203 (27.8%)
Mode of delivery	
Vaginal delivery	431 (59.1%)
Cesarean delivery	296 (40.6%)
Family income (CNY)	
<2000	25 (3.4%)
2000–4999	376 (51.5%)
5000–9999	276 (37.8%)
>10,000	31 (4.2%)
Mother’s education	
≤12 years	103 (14.1%)
>12years	617 (84.5%)
Father’s education	
≤12 years	100 (13.7%)
>12years	622 (85.2%)
Paternal smoking during pregnancy	
yes	385 (52.7%)
no	324 (44.4%)
Parenting quality	
Good parenting quality	224 (33.6%)
Poor parenting quality	443 (66.4%)

Note: Frequencies may not equal 730 due to rounding and missing responses for some questions.

**Table 2:** Effects of parenting quality on children’s development outcome.

		Parenting Concepts	Attention	Parent–Child Relationship	Learning Material	Language Stimulation	Outside Activity	Feeding	Safety
CM	*β*	−0.57	−0.05	−0.09	−0.97	−0.47	−1.00	−0.03	0.11
	*β^a^*	−0.58	−0.01	−0.26	−0.11	−1.48	−0.44	−0.30	−0.48
	*p*	0.004 **	0.971	0.007 **	0.551	0.000 ***	0.104	0.053	0.000 ***
GM	*β*	0.28	−0.66	−0.18	−0.99	−0.78	−0.96	0.25	−0.07
	*β^a^*	0.17	−0.92	−0.54	−0.46	−0.91	−0.33	0.01	−0.14
	*p*	0.321	0.000 ***	0.000 ***	0.010 *	0.023 *	0.128	0.960	0.112
FM	*β*	−0.96	0.39	0.22	−1.44	0.94	−0.03	−0.71	0.42
	*β^a^*	−0.95	0.50	0.20	−1.36	0.73	−0.10	−0.63	−0.43
	*p*	0.021 *	0.368	0.448	0.003 **	0.359	0.864	0.077	0.085
CG	*β*	−0.28	−0.09	0.07	0.01	0.24	0.40	−0.43	0.01
	*β^a^*	0.28	−0.05	0.05	0.09	0.28	0.33	−0.42	−0.01
	*p*	0.349	0.889	0.719	0.777	0.566	0.346	0.050 ^*^	0.971
PS	*β*	0.36	−1.37	−0.42	−0.56	1.04	−0.78	0.04	−0.34
	*β^a^*	0.33	−1.35	−0.42	−0.51	0.93	−0.83	0.10	−0.34
	*p*	0.277	0.002 *	0.041 *	0.172	0.123	0.032 *	0.695	0.094

*β*: regression coefficient; *β^a^*: adjusted regression coefficient; CM: communication; GM: gross motor; FM: fine motor; CG: problem-solving; PS: personal-social. Level of significance: * *p* < 0.05, ** *p* < 0.01, *** *p* < 0.001. GEE was used to assess the effects. The dependent and independent variables were quantitative variables, while confounders were qualitative variables. Dependent variables were the scores of CM, GM, FM, CG, and PS. *β^a^* was adjusted for child gender, family income, parental education, delivery mode, and child order.

**Table 3:** Factors affecting suspected development delay.

	Suspected Development Delay OR (95% CI)	*p*
Parenting quality		
Good parenting quality	1.00 (reference)	-
Poor parenting quality	2.74 (1.17, 6.40)	0.020 *
Child gender		
Boy	1.00 (reference)	-
Girl	0.33 (0.16, 0.69)	0.003 *
Child order		
<2	1.00 (reference)	-
≥2	1.52 (0.66, 3.48)	0.323
Mode of delivery		
Vaginal delivery	1.00 (reference)	-
Cesarean delivery	0.96 (0.48, 1.93)	0.905
Family income (CNY)		
<2000	1.00 (reference)	-
2000–4999	1.53 (0.25, 9.28)	0.282
5000–9999	1.60 (0.27, 9.68)	0.608
>10,000	3.68 (0.34,39.56)	0.643
Mother’s education		
≤12 years	1.00 (reference)	-
>12years	0.27 (0.12, 0.64)	0.003 *
Father’s education		
≤12 years	1.00 (reference)	-
>12years	1.60 (0.58, 4.40)	0.371
Paternal smoking during pregnancy		
yes	1.00 (reference)	-
no	1.29 (0.64, 2.60)	0.470

OR: odds ratios; CI: confidence interval; Level of significance: * *p* < 0.05. General linear models were used to assess the effects. The dependent and independent variables were qualitative variables.
